# Outcomes of microaxial flow pump use in acute-on-chronic heart failure

**DOI:** 10.1016/j.jhlto.2026.100532

**Published:** 2026-03-10

**Authors:** W.H. te Gussinklo, T. Azami, G. Romano, M. Eden, P. Schlegel, J. Kremer, R. Arif, M. Karck, A.L. Meyer

**Affiliations:** a Department of Cardiac Surgery, University Hospital Heidelberg, Heidelberg, Germany; b Department of Cardiology, University Hospital Heidelberg, Heidelberg, Germany

**Keywords:** Microaxial flow pump, Acute heart failure, Chronic heart failure, Temporary ventricular assist device, Mechanical circulatory support

## Abstract

**Background:**

The mortality rate associated with acute decompensated heart failure (ADHF) is high, and prognosis depends on restoring cardiac output. Patients frequently require mechanical circulatory support (MCS). The Impella microaxial flow pump (mAFP) is a short-term MCS used to stabilize patients. We report outcomes of mAFP use in patients with pre-existing chronic heart failure who presented with ADHF.

**Materials and Methods:**

This retrospective single-center study included all patients with a history of chronic heart failure who presented with ADHF and were managed with mAFP. Demographic characteristics, hemodynamic variables, complications, and clinical outcomes were evaluated.

**Results:**

Fifty-eight patients were included. Patients were managed with the Impella 5.5/5.0 (*n* = 52, 89.7%) or CP (*n* = 6, 10.3%). After mAFP implantation, there was a significant reduction in vasoactive inotropic score (*p* < 0.001), increase in glomerular filtration rate (*p* < 0.001), and reduction in lactate levels (*p* < 0.001). Two patients (3.4%) were lost to follow-up, 28 (51.2%) were bridged to durable left ventricular assist device (LVAD), and 3 (5.2%) were bridged to heart transplant. Eight patients (13.8%) were successfully weaned; 17 (29.3%) died while receiving mAFP support. Major complications included bleeding (*n* = 23, 39.7%), local infection (*n* = 37, 63.8%), sepsis (*n* = 9, 15.5%), and renal dysfunction (*n* = 34, 58.6%).

**Conclusion:**

The mAFP demonstrates favorable outcomes in patients with chronic heart failure who present with ADHF. It resulted in significant improvements in hemodynamic parameters, and most patients survived to durable LVAD or transplantation. In this heterogenous patient group, only a few survived definitive weaning from mAFP.

## Background

Acute decompensated heart failure (ADHF) is marked by the sudden onset of symptoms such as dyspnea, fatigue, and fluid retention. In most cases, it represents a critical phase in the progression of chronic heart failure.^1^ It is associated with considerable heterogeneity in outcomes, with an estimated mortality rate of up to 43%.^2^ Moreover, increased survival among patients with myocardial infarction and heart failure has contributed to a growing population of patients with ADHF.^3^ Treatment strategies for ADHF differ from those used in chronic heart failure, prioritizing rapid symptom relief and hemodynamic stabilization rather than long-term remodeling and mortality reduction.

Initial management typically involves pharmacologic therapy; however, many patients fail to respond adequately and require mechanical support. In such instances, short-term mechanical circulatory support (MCS) devices are vital for maintaining perfusion and may serve as a bridge to transplantation or durable MCS. These temporary ventricular assist devices include the intra-aortic balloon pump, extracorporeal life support (ECLS), and microaxial flow pump (mAFP).^4^

The Impella 5.5/5.0 or CP device (Johnson & Johnson, Danvers, MA, USA) is a microaxial left ventricular assist device providing temporary MCS by directly unloading the left ventricle. This action reduces pulmonary congestion and increases cardiac output.^5^, ^6^ Due to its minimally invasive design and rapid deployment, the Impella is well suited for patients with hemodynamic instability.

This study aims to analyze outcomes associated with mAFP use in patients with ADHF and chronic heart failure, as there is a relative paucity of data regarding this specific patient group. By examining a range of clinical variables, we assess the clinical results, complications, and further clinical course of these patients after mAFP therapy. Additionally, we contextualize our findings within the literature on mAFP and other commonly used MCS devices and add to the growing body of evidence that mAFP is the first-choice treatment as bridge-to-decision in this heterogenous patient group in end-stage heart failure.^7^

## Materials and methods

### Study design and population

This retrospective study included all patients between 2019 and 2023 with a history of chronic heart failure who presented to our hospital with ADHF and were managed with an mAFP. The study protocol was approved by the institutional review board, and the requirement for informed consent was waived due to the retrospective design.

Patients were excluded if they had incomplete records, recent myocardial infarction, or post-cardiotomy syndrome.

Chronic heart failure was defined as documented reduced left ventricular function before admission with guideline-directed therapy for ≥6 weeks. ADHF was diagnosed based on clinical assessment and confirmed by echocardiography showing reduced output.

All patients received temporary MCS with an mAFP device (Impella CP, Impella 5.5, or 5.0) in addition to standard medical therapy. Device use was indicated due to inadequate response to inotropic support and the need for hemodynamic support. All patients were classified as INTERMACS 2 or 1 patients. First line therapy in ADHF in chronic heart failure patients who do not respond adequately to inotropic support in our institution is the use of the Impella 5.0/5.5. The decision to escalate to mAFP is made taking into consideration urine output, increasing lactate levels, central venous pressure, and increasing need of inotropic support.^8^ If there is right ventricular failure as well, the use of ECLS is also indicated, and mAFP and ECLS are used simultaneous. The Impella CP was used in patients who were not eligible for Impella 5.5. implantation due to vascular status or due to emergency implantation in the cath lab.

### Data collection

Collected demographic data included age, sex, body mass index, comorbidities, and medications administered prior to admission. Peri-implant variables comprised echocardiographic assessments before and after device placement, laboratory parameters (blood counts, renal and liver function tests, arterial blood gas, vasoactive-inotropic score (VIS), and implant details (duration, access site, and device type). The presence of additional cardiac devices was also recorded. Outcome measures included successful weaning, transition to destination therapy, or death, along with device-related complications. Bleeding events were categorized per INTERMACS adverse event definitions.^9^ Successful weaning was defined as survival ≥30 days after device explantation without further mechanical support.

### Statistical analysis

Statistical analyses were conducted using IBM SPSS Statistics for Windows, version 25 (IBM Corp., Armonk, NY, USA). Descriptive statistics summarized the data; numerical variables were reported as mean, median, and standard deviation, and categorical variables as frequency and percentage. Normality was assessed using skewness and kurtosis. When data were not normally distributed, non-parametric tests were employed.

Differences between pre- and post-implant laboratory results and arterial blood gas were analyzed using the Wilcoxon signed-rank test. Changes in catecholamine VIS over time were evaluated by Friedman’s analysis of variance, followed by post hoc testing when applicable. Associations between outcomes and clinical characteristics were assessed using Spearman’s rank correlation. Logistic regression analysis was performed to predict the likelihood of outcome variables.

## Results

### Patient demographics and baseline characteristics

A total of 58 patients with ADHF were included. The mean age was 50.7 ± 12.2 years, with 70.7% (*n* = 41) being male. The mean body mass index was 26.5 ± 6.4 kg/m^2^. Primary etiologies were dilated cardiomyopathy (*n* = 40, 69.0%), ischemic cardiomyopathy (*n* = 6, 10.3%), hypertrophic cardiomyopathy (*n* = 4, 6.9%), post myocarditis (*n* = 6, 10.3%), sarcoidosis (*n* = 1, 1.7%), and other causes (*n* = 1, 1.7%). All patients had a documented history of chronic heart failure and developed ADHF upon presentation to the emergency department. Demographic details, comorbidities, and clinical history are summarized in Table 1.

**Table 1:** Baseline Characteristics

	Total *n* = 58	Impella 5.0/5.5 *n* = 52 (89.7%)	Impella CP *n* = 6 (10.3%)
Gender (male)	41 (70.7%)	38 (73.1%)	3 (50.0%)
Age (years)	50.7±12.0	50.8±12.3	52.0±10.9
BMI (kg/m²)	26.5±6.4	25.8±5.77	32.4±9.7
*Preexisting cardiac disease*			
− DCM − ICM − HCM − Post-myocarditis	40 (69%) 6 (10.3%) 4 (6.9%) 6 (10.3%)	38 (73.1%) 6 (11.5%) 3 (5.8%) 5 (9.6%)	2 (33.3%) 0 1 (16.7%) 1 (16.7%)
− Sarcoidosis − Other	1 (1.7%) 1 (1.7%)	0 0	1 (16.7%) 1 (16.7%)
Hypertension	25 (43.1%)	20 (38.5%)	5 (83.3%)
Diabetes	11 (19.0%)	10 (19.2%)	1 (16.7%)

Abbreviations: BMI, Body mass index; DCM, Dilated cardiomyopathy; ICM, Ischaemic cardiomyopathy; HCM, Hypertrophic cardiomyopathy.

### Implant device utilization

The mAFP was used for an average of 12.8 ± 9.7 days. Most patients received the Impella 5.5/5.0 (*n* = 52, 89.7%), while a smaller group received the Impella CP (*n* = 6, 10.3%). The right subclavian artery was the most common vascular access site (*n* = 53, 91.4%), followed by the right femoral artery (*n* = 5, 8.6%). The mAFP was used concurrently with VA-ECLS in 17.2% (*n* = 10) of patients.

### Clinical outcomes

The mean intensive care unit stay was 32.2 ± 35.3 days. Following mAFP explantation, 28 patients transitioned to durable left ventricular assist devices (dLVAD, *n* = 28, 48.3%), and three underwent heart transplantation (*n* = 3, 5.2%). Seventeen patients (29.3%) died while receiving Impella support, and 8 (13.8%) met criteria for successful weaning, of whom 5 survived ≥1-year post-explantation. Data were unavailable for two patients. Of the 17 patients that died, 13 (76.5%) died from cardiogenic shock despite mAFP implantation, and 4 (23.5%) died due to multiorgan failure. Bleeding events during mAFP support were classified as type 1 (*n* = 1, 1.7%), type 2 (*n* = 17, 29.3%), type 3 (*n* = 4, 6.9%), and type 4 (*n* = 1, 1.7%). Eight patients (13.8%) required transfusions. The patient with type 4 bleeding developed a local hematoma requiring surgical drainage. Major complications included pneumonia (*n* = 37, 63.8%), sepsis (*n* = 9, 15.5%), and renal dysfunction (*n* = 34, 58.6%). These are detailed in Table 2.

**Table 2:** Clinical Outcomes

Parameter	Number of patients *n* = 58
30-day survival	41 (70.7%)
Successfully weaned	8 (13.8%)
Died on Impella support	17 (29.3%)
Bridge to dLVAD	28 (48.3%)
Bridge to heart transplantion	3 (5.2%)
ICU stay (days)	32.2±35.3
Bleeding duringmAFP support (INTERMACS)	
− 1 − 2 − 3 − 4 − 5	35 (60.3%) 1 (1.7%) 17 (29.3%) 4 (6.9%) 1 (1.7%)
Infection	37 (63.8%)
Sepsis	9 (15.5%)
AKI	34 (58.6%)
Respiratory failure	15 (25.9%)
Cardiac arrhythmia	10 (17.2%)
Pericardial fluid collection	3 (5.2%)
Limb ischemia	2 (3.4%)
Haemorrhagic stroke	4 (6.9%)
Ischemic stroke	0

Abbreviations: dLVAD, durable Left Ventricular Assist Device; AKI, Acute Kidney Injury.

### Hemodynamic improvement

Significant improvements in hemodynamic parameters were observed after mAFP implantation. All patients had left ventricular ejection fraction <35% on pre-implant echocardiography. Changes in left ventricular function post-explant could not be consistently assessed, preventing statistical analysis. However, significant differences in pre- and post-mAFP VIS score (42.3 ± 56.0 vs 17.5 ± 20.6, *p* = 0.001), glomerular filtration rate (43.7 ± 23.5 mL/min/1.73 m² vs 64.3 ± 37.9 mL/min/1.73 m², *p* < 0.001), and lactate levels (45.5 ± 28.5 mg/dL vs 17.2 ± 22.8 mg/dL, *p* < 0.001) indicated hemodynamic improvement and improvement in end-organ function. These findings are summarized in Table 3.

**Table 3:** Changes in Hemodynamic Parameters Before and After mAFP Implantation

	Pre-implant	Post-implant	*p*-value
VIS score	42.3±56.0	17.5±20.6	0.001
GFR (mL/min/1.73 m²)	43.7±23.5	64.3±37.9	<0.001
Lactate (mG/dL)	45.5±28.5	17.2±22.8	<0.001
Bilirubin (mG /dL)	3.70±4.33	7.77±10.70	0.112

### Survival analysis

The overall 30-day survival rate was 70.7% (*n* = 41). Among survivors, 31 patients transitioned to destination therapy, while 8 remained hemodynamically stable 30 days after mAFP explantation. In the Impella 5.5/5.0 group 39 (75%) survived, whereas in the Impella CP group 2 (33%) survived. This data suggests improved survival among patients receiving the Impella 5.5/5.0 compared to those with the Impella CP; however, the small CP subgroup size and skewed data distribution precluded comparative statistical analysis. Therefore, no definitive conclusions could be drawn regarding device-specific survival differences.

## Discussion

The management of ADHF remains a significant clinical challenge, particularly in patients with chronic heart failure who present with hemodynamic instability unresponsive to medical therapy. An mAFP, as temporary MCS, provides an opportunity to stabilize these patients by delivering direct mechanical cardiac support.^10^ This retrospective study offers a comprehensive analysis of outcomes associated with mAFP use in patients with ADHF and chronic heart failure.

ADHF involves a marked decline in cardiac output, triggering a cascade of pathophysiological processes. This decline causes venous congestion and systemic inflammation, leading to the release of vasoactive mediators. A key consequence is impaired organ perfusion, frequently resulting in acute kidney injury.^11^ Implantation of an mAFP as non-durable MCS in patients with ADHF has been associated with improved cardiac output and mitigation of these events.^12^, ^13^, ^14^

The DanGer Shock trial provided the first evidence supporting Impella in cardiogenic shock. Patients with ST-elevated myocardial infarction complicated by cardiogenic shock exhibited reduced all-cause mortality at 180 days when treated with mAFP. Although the mAFP group faced a higher risk of composite adverse events,^15^ the study demonstrated that improving cardiac output in patients with cardiogenic shock increased survival.

Our findings align with prior reports. Although direct measurements of cardiac output were unavailable due to incomplete records, we observed significant reductions in VIS scores and lactate levels, along with improved glomerular filtration rate. Earlier studies describe reductions in pulmonary congestion and enhanced gas exchange under mAFP support, which may help explain the benefits observed in our cohort.^16^

All patients in our cohort had an ejection fraction <35% before mAFP implantation, necessitating prompt mechanical support given limited pharmacologic options. Our results also support evidence that early mAFP use is critical to prevent progression to multi-organ failure and to improve prognosis.^17^, ^18^

We included patients with ADHF and chronic heart failure, most with non-ischemic cardiomyopathy. For analysis, patients with either the Impella 5.0/5.5 or Impella CP devices were grouped. Although the number of patients with the CP device was too small for definitive statistical comparisons, clinical trends suggest that the Impella 5.5/5.0 may be preferable in this population due to higher flow and greater augmentation of cardiac output.^19^ Notably, none of the patients supported by the Impella CP device were bridged to durable LVAD therapy, and this group experienced a mortality rate of 66.7%. Despite the limited sample size, these observations support the hypothesis that higher-output devices may offer superior hemodynamic support in patients with advanced chronic heart failure presenting with ADHF. Further studies with larger cohorts are needed to validate this finding.

Hong et al^20^ analyzed 137 patients with cardiogenic shock treated with the Impella 5.0/5.5. They included patients with acute MI (*n* = 47), ADHF (*n* = 86), and postcardiotomy (*n* = 4). The ADHF group had the highest survival rate, with nearly two-thirds alive at 180 days. Only 17% of patients were weaned from mAFP support, while most were bridged to durable LVAD therapy or transplantation. In contrast, more than half of patients requiring mAFP after myocardial infarction were successfully weaned. A similar pattern emerged in our cohort, with only eight patients (13.8%) successfully weaned, while 31 (53.4%) were bridged to durable LVAD implantation or transplantation. The patients who were successfully weaned were not characterized by common features, and weaning should be decided on a case-to-case basis. Only 5 (8.6%) patients initially meeting weaning criteria were alive after one year. These findings underscore that in patients with acute-on-chronic heart failure requiring mAFP, support should be implemented as bridge-to-bridge or destination therapy.

The overall survival rate in our study population was 70.7% (*n* = 41), comparable to findings by Fried et al,^21^ who reported outcomes in patients with cardiogenic shock secondary to myocardial infarction or decompensated heart failure. They observed an in-hospital survival of 73.6% and were able to wean 17% of patients from mAFP support, while 57% were bridged to transplantation or durable LVAD therapy. However, their cohort also included patients with de novo heart failure, differing from our exclusively chronic heart failure population. In our cohort, only a small proportion of patients underwent heart transplantation, primarily due to the limited regional availability of donor organs.

Complications associated with the device in our study included bleeding, infection, sepsis, limb ischemia, and renal dysfunction. These align with the inherent risks of all MCS devices.^22^ To our knowledge, this case series is the first dedicated analysis of mAFP outcomes in patients with chronic heart failure and acute decompensation. Previous studies assessed mixed cohorts with diverse etiologies necessitating mAFP.

Bleeding remains one of the most significant complications of mAFP, with reported rates ranging between 15% and 25%.^23^, ^24^ In our study, bleeding events were classified using INTERMACS adverse event definitions. The most frequent complication was type 2 bleeding (*n* = 17, 29.3%), characterized by overt bleeding requiring escalated care and further evaluation. Type 3 bleeding occurred in four patients (6.9%), defined by an acute hemoglobin drop necessitating transfusion. Only one patient (1.7%) experienced type 4 bleeding requiring surgical intervention. Although bleeding was controlled with pressure, the patient developed a large hematoma requiring surgical drainage. No cases of fatal bleeding (type 5) were observed.

Bleeding complications were more frequent among older adults and female patients. This may reflect the higher prevalence of peripheral arterial disease in older adults and smaller vessel caliber in females.^24^ Although the Impella CP has been associated with fewer vascular and bleeding complications,^25^ our CP group was too small to confirm this observation. It is recommended that patients with Impella devices undergo regular monitoring for hemolysis. Esposito et al^25^ advise frequent assessment of bilirubin, haptoglobin, and lactate dehydrogenase every 6-12 hours to facilitate early detection and prevent complications such as acute kidney injury. In our series, pre- and post-mAFP values of LDH and bilirubin did not differ significantly. However, the absence of standardized timing for post-implantation measurements and lack of follow-up data limits interpretation.

It is essential to consider ECLS, the current standard of care for patients with ADHF requiring MCS. Unlike mAFP, ECLS supports circulation from the right atrium to the aorta.^26^ Although ECLS reduces left ventricular end-diastolic pressure and volume, studies indicate it may be less effective in patients with severe heart failure.^27^

A case series by Garan et al,^28^ including 52 patients with dilated cardiomyopathy managed with ECLS for ADHF, showed that only 46.2% (*n* = 24) survived to hospital discharge, and 19 patients (36.5%) were bridged to LVAD therapy. In contrast, 41 patients (70.7%) in our cohort survived after mAFP support, and 28 (48.3%) were successfully bridged to durable LVAD implantation or transplantation. This comparison suggests a higher survival rate among patients with acute-on-chronic heart failure supported by mAFP therapy.

In our series, ten (17.2%) patients were managed with simultaneous ECLS and mAFP to enhance gas exchange and augment cardiac output. This strategy also offers flexibility: once gas exchange is sufficiently restored, ECLS can be discontinued safely, while mAFP is maintained to provide ongoing left ventricular support.^29^ In this cohort, the mortality rate was 50% (*n* = 5), likely reflecting the worse clinical condition necessitating ECMO due to lung failure or right ventricular failure.

This study has several limitations. First, its retrospective design limited the availability of clinical variables, as the condition often arises as a medical emergency, making it difficult to collect data retrospectively. Second, the relatively small sample size and single-center design reduces the statistical power and limits the generalizability of the results to broader patient populations. Additionally, management and device selection in this heterogenous patient group was difficult to standardize. Lastly, the lack of a control or comparison group hinders definitive conclusions regarding the specific benefits of mAFP in this context.

## Conclusion

This study highlights the potential benefits of mAFP MCS in improving hemodynamic stability in patients with ADHF and chronic heart failure. It also emphasizes the potential risks and the importance of meticulous management when using this advanced MCS approach. Compared to outcomes reported for ECLS, mAFP use in this patient population was associated with higher rates of successful conversion to destination therapy, improved survival, and fewer complications. Notably, our cohort demonstrated that only a small percentage of patients can be successfully weaned, and most will require destination therapy. Underlying the necessity to immediately start planning for a treatment plan after stabilizing and mAFP use. This small heterogenous cohort has shown promising results in managing acute-on-chronic heart failure with an mAFP, but future prospective, randomized controlled trials are necessary to refine treatment strategies and validate these findings in this critically ill population.

## Disclosure statement

A.L. Meyer has received lecture fees from Johnson & Johnson, Danvers, MA, USA.

## Conflicts of Interest statement

The authors declare the following financial interests/personal relationships, which may be considered as potential competing interests: Anna Meyer reports a relationship with Johnson & Johnson MedTech that includes: speaking and lecture fees. If there are other authors, they declare that they have no known competing financial interests or personal relationships that could have appeared to influence the work reported in this paper.
